# Supplementary material for: Beneficial Effect of ACI-24 Vaccination on Aβ Plaque Pathology and Microglial Phenotypes in an Amyloidosis Mouse Model
Source: Cells. 2022 Dec 24;12(1):79. doi: 10.3390/cells12010079 (PMC9818422; doi:10.3390/cells12010079)
Supplement: Supplementary file 1 [file cells-12-00079-s001.zip › cells-2065587-supplementary.pdf]

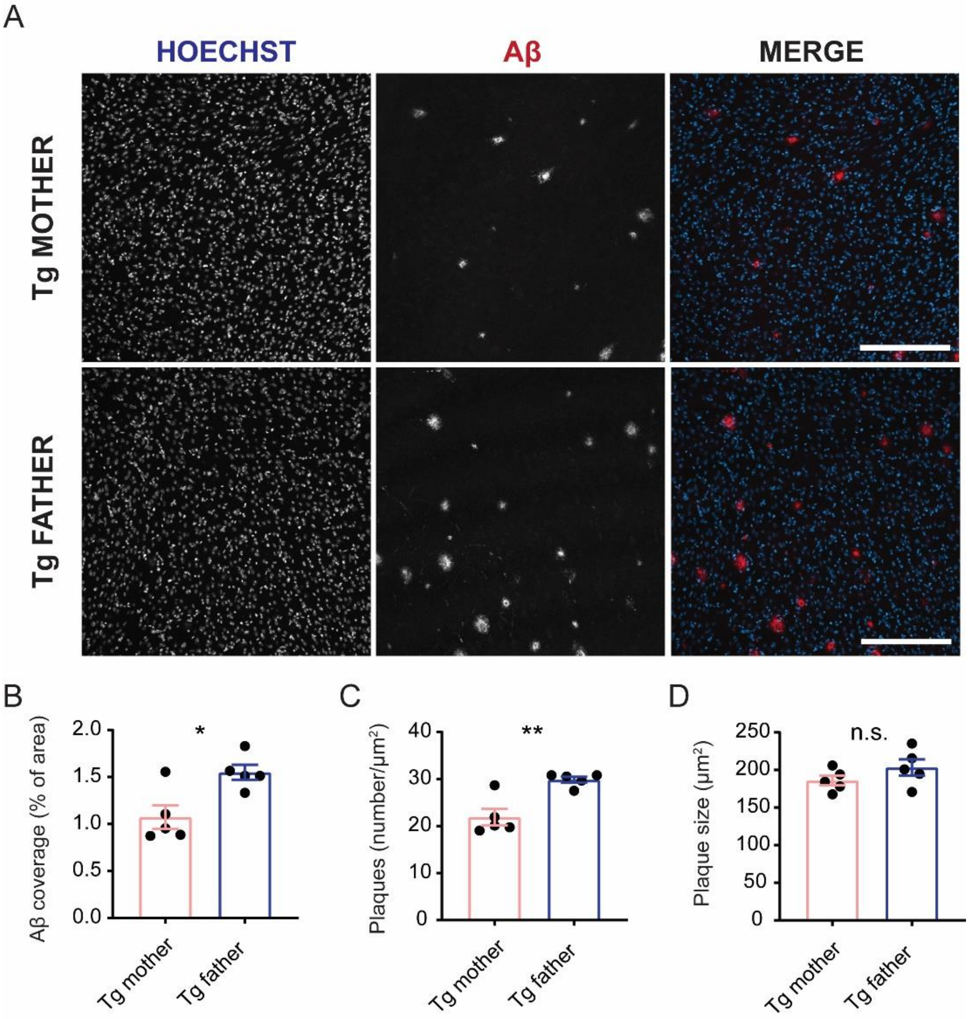

Figure S1. Higher Aβ plaque load in transgenic father compared to transgenic mother offspring**.** Representative images of control treated mice (transgenic mother and father offspring) stained for nuclei (Hoechst) and Aβ (NAB228 antibody) (A). Scale bar: 200 µm. Statistical analysis of Aβ plaque coverage (B), number (C) and size (D). ACI-24 demonstrates increase in Aβ plaque coverage and number in transgenic father (blue) compared to transgenic mother offspring (pink). Graphs are presented as mean ± SEM (n.s. non-significant, *p<0.05, **p< 0.001, unpaired two-tailed Student's T-test).

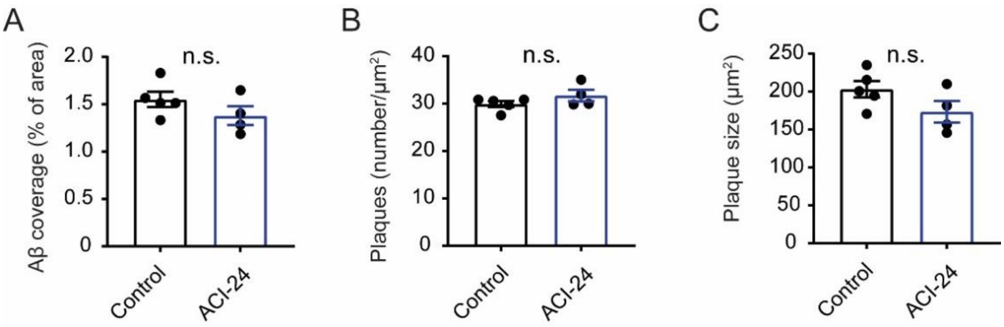

Figure S2. ACI-24 has no major impact on Aβ plaque pathology in the offspring generated by the transgenic father. Statistical analysis of 5 control and 4 ACI-24 vaccinated mice (transgenic father offspring) for Aβ plaque coverage (B), number (C) and size (D). Graphs are presented as mean ± SEM (n.s. non-significant, unpaired two-tailed Student's T-test).

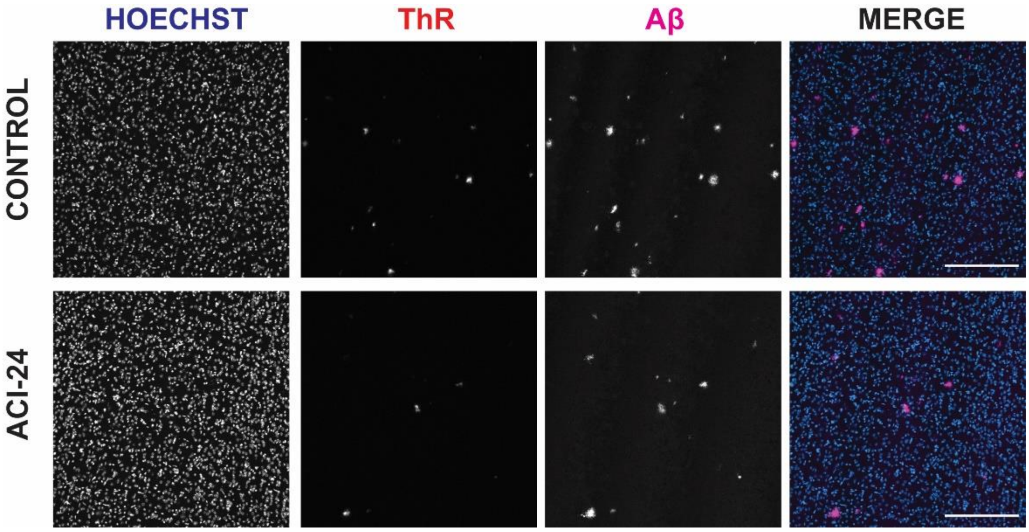

Figure S3. ACI-24 reduces plaque pathology in the transgenic mother offspring. Representative images of 5 control and 6 ACI-24 vaccinated mice (transgenic mother offspring) for nuclei (Hoechst), fibrillar Aβ (ThR) and Aβ plaques (NAB228 antibody). Scale bar: 200 μm.

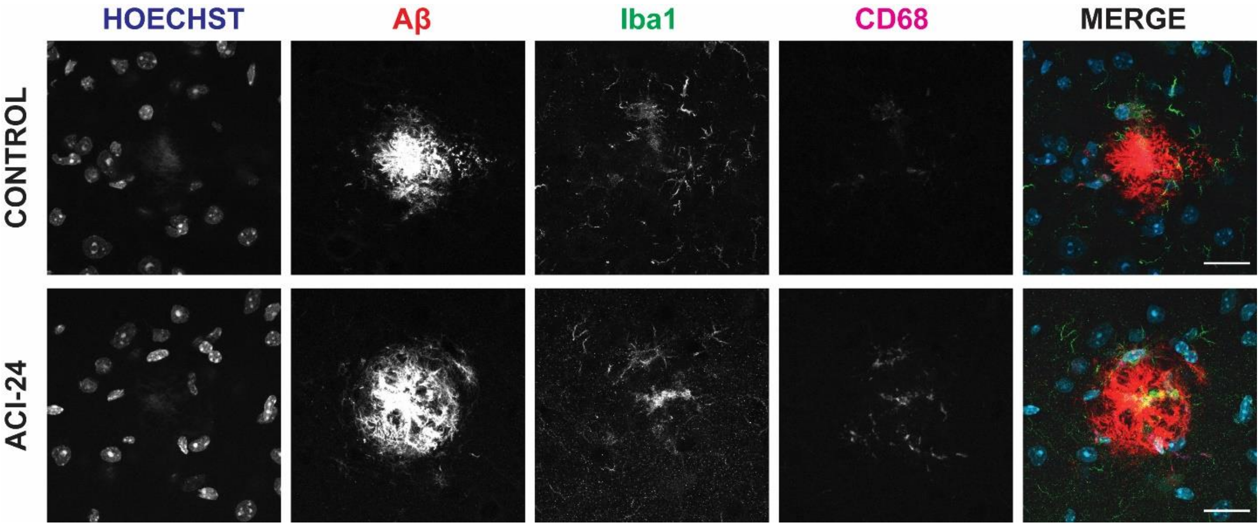

Figure S4. ACI-24 shows a trend towards increased microglial activation at the Aβ plaque in the transgenic mother offspring. Representative images of 5 control and 6 ACI-24 vaccinated mice (transgenic mother offspring) for nuclei (Hoechst), Aβ plaques (3552 antibody) and microglia (Iba1 and CD68). Scale bar: 20 μm.

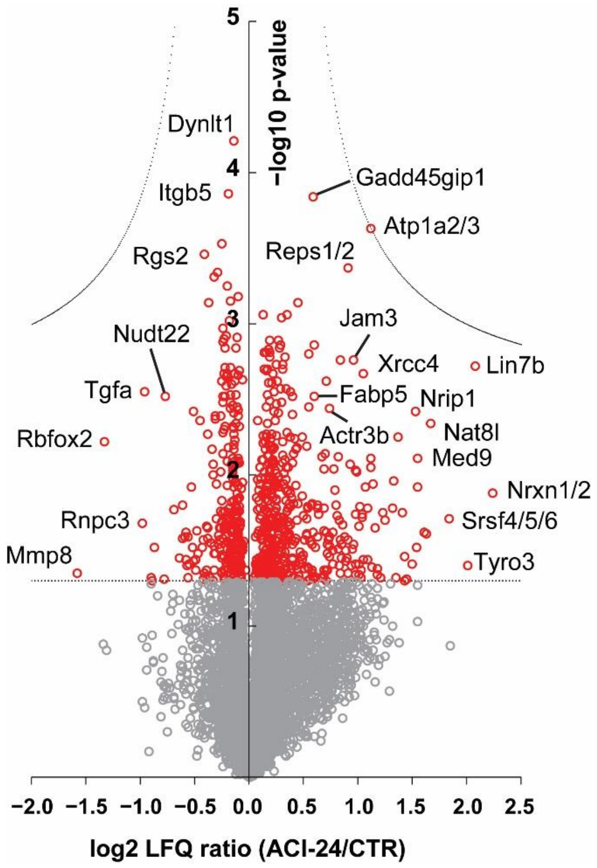

Figure S5. No major changes in microglial proteome following ACI-24 vaccination. Volcano plot of acutely isolated microglia from cerebrum of 5 control (3 transgenic father and 2 transgenic mother offspring) and 6 ACI-24 vaccinated mice (3 transgenic father and 3 transgenic mother offspring). The negative log10 transformed p-value is plotted against the mean protein log2 transformed ratio between ACI-24 vaccinated and control mice. Dotted line represents the p-value of 0.05, while the hyperbolic curve indicates the threshold of a permutation-based FDR correction for multiple hypotheses (FDR <0.05, s0= 0.1). Non-significant proteins are marked in grey circles, while the proteins with p-value less than 0.05 are marked in red circles. None of the protein changes are significant according to the FDR correction.

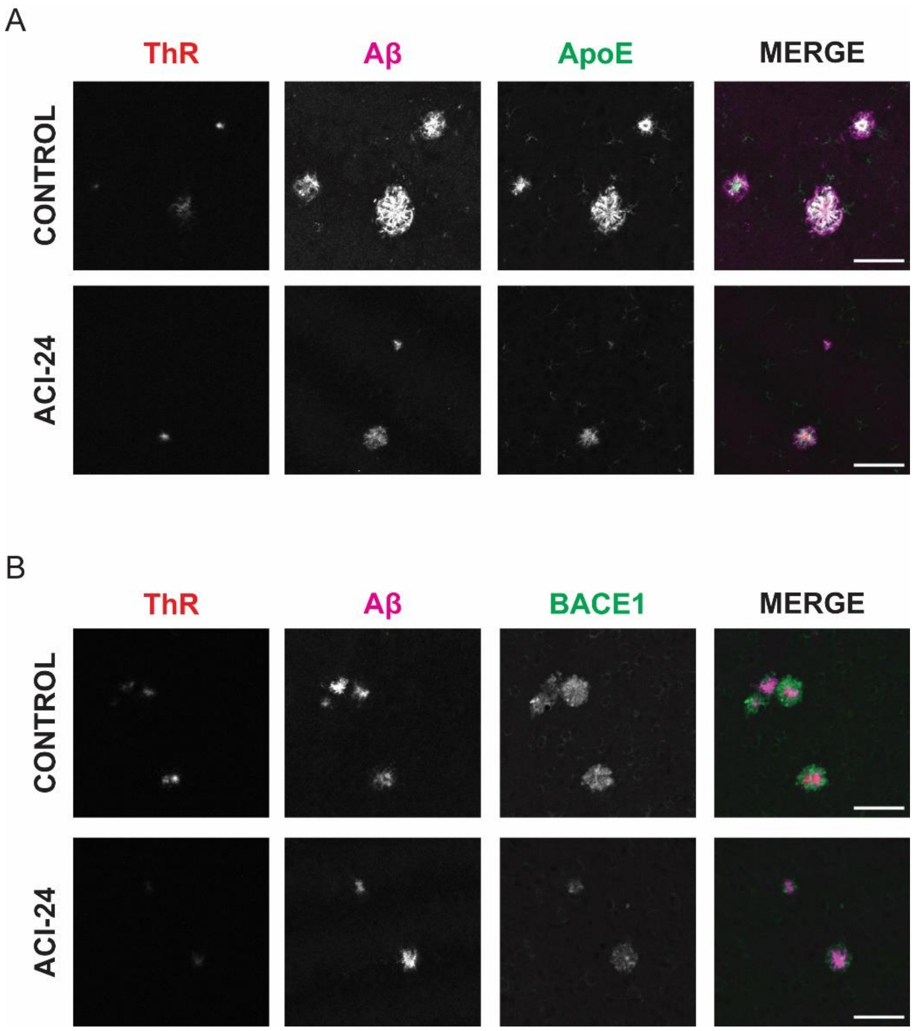

Figure S6. ACI-24 reduces ApoE protein levels and neuronal injury in the transgenic mother offspring. Representative zoomed-in images of 5 control and 6 ACI-24 vaccinated mice (transgenic mother offspring) for fibrillar Aβ (ThR), Aβ plaques (3552 antibody) and ApoE (A); and fibrillar Aβ (ThR), Aβ plaques (NAB228 antibody) and BACE1 (B). Scale bar: 50 μm.

Probe Name	Accession #	Ratio (ACI vs. PBS)	P value
Cst7	NM_009977.3	0.45	0.02624886
Cd163	NM_053094.2	0.76	0.02575657
Cd4	NM_013488.2	0.77	0.01494986
Trem2	NM_031254.2	0.79	0.00094278
Ggta1	NM_010283.4	0.79	0.02090645
Bhlhe41	NM_024469.1	0.8	0.03066171
Casp3	NM_009810.2	0.82	0.03023026
Cd68	NM_001291058.1	0.83	0.01253124
Tgfbr1	NM_009370.2	0.84	0.03861323
Fcrls	NM_030707.3	0.86	0.00997866
Slco2b1	NM_001252531.1	0.86	0.04334012
Usp2	NM_016808.2	0.86	0.0461054
Jun	NM_010591.2	0.88	0.02938688
Stat2	NM_019963.1	0.88	0.03495526
Aif1	NM_019467.2	0.89	0.03693615
Slc2a5	NM_019741.3	0.9	0.00049585
Siglech	NM_178706.4	0.9	0.00853947
C1qa	NM_007572.2	0.9	0.01303327
C1qc	NM_007574.2	0.91	0.0162393
Grn	NM_008175.4	0.91	0.0193998
Olfml3	NM_133859.2	0.92	0.03104119
C1qb	NM_009777.2	0.92	0.0327872
Hexb	NM_010422.2	0.93	0.006605
Grm1	NM_001114333.2	0.93	0.03968352
Cx3cr1	NM_009987.4	0.94	0.0326831
Cst3	NM_009976.3	0.95	0.03138557
Hdac2	NM_008229.2	0.97	0.02670269
Ube2n	NM_080560.3	1.02	0.03309815
Grk3	NM_177078.4	1.09	0.0418932
Arntl	NM_007489.3	1.17	0.01129997
Etv5	NM_023794.2	1.18	0.03231741

Table S1. Table of differentially expressed genes using Glial Profiling Panel. Significantly changed genes according to the p-value of 0.05 (non-FDR-corrected) analysed with Glial Profiling Panel (NanoString) including 5 control and 6 ACI-24 vaccinated mice (transgenic mother animals) are shown with the gene name, accession number, ratio and p-value.

Probe Name	Accession #	Ratio (ACI vs. PBS)	P value
Il10ra	NM_008348.2	0.75	0.001589
Naglu	NM_013792.2	0.8	0.000842
Sp100	NM_013673.3	0.8	0.006672
Psmb8	NM_010724.2	0.8	0.044439
Epha3	NM_010140.3	0.8	0.048772
Cd68	NM_009853.1	0.82	0.034259
Tgfb1	NM_011577.1	0.86	0.030453
Rela	NM_009045.4	0.86	0.049765
Cyp4x1	NM_001003947.1	0.9	0.00924
Dgkb	NM_178681.4	0.9	0.048782
Aldh1l1	NM_027406.1	0.91	0.034743
Hpgds	NM_019455.4	0.94	0.020486
Ccr5	NM_009917.5	1.54	0.002537

Table S2. Table of differentially expressed genes using Neuropathology Panel. Significantly changed genes according to the p-value of 0.05 (non-FDR-corrected) analysed with Neuropathology Panel (NanoString) including 5 control and 6 ACI-24 vaccinated mice (transgenic mother animals) are shown with the gene name, accession number, ratio and p-value.
